# Teaching in times of COVID-19. Challenges and opportunities for digital teaching

**DOI:** 10.3205/zma001396

**Published:** 2020-12-03

**Authors:** Daniel Tolks, Sebastian Kuhn, Sylvia Kaap-Fröhlich

**Affiliations:** 1 Klinikum der LMU München, Institut für Didaktik und Ausbildungsforschung in der Medizin, München, Germany; 2 Leuphana Universität Lüneburg, Zentrum für angewandte Gesundheitswissenschaften, Lüneburg, Germany; 3 Universität Bielefeld, Med. Fakultät OWL, AG 4 Digitale Medizin, Bielefeld, Germany; 4 Careum Bildungsmanagement, Zürich, Switzerland

## Editorial

The year 2020. Nobody was really prepared for the radical changes and challenges that year. The COVID-19 pandemic suddenly forced hospitals and clinics, but also schools and universities, to find new ways to maintain both supply and teaching and learning. This process had to be quick and often led to the much-cited term “Emergency Remote Teaching” [1]. But we also realised that digital education and training could and had to show what potential it had. 

Before the COVID-19 pandemic there were already very positive developments in digital learning and teaching. There was already a lot of evidence in basic research that could highlight the advantages of the targeted use of digital teaching [2], [3], [4], [5], [6]. New technologies and teaching methods as well as well-founded explanatory models, such as the ICAP model or e-activities, have been iteratively developed further and represented a selective enrichment of the teaching offer at a number of institutions [7], [8], [9]. In particular blended learning concepts such as the Inverted Classroom Model, but also the use of virtual patients and simulations were increasingly used [10], [11], [12], [13]. However, there were also relevant hurdles that slowed down digital teaching. A lack of experience on the part of teachers, legal framework conditions at various levels of health and education policy [14], [15], a lack of transparency in the creditability of digital teaching and other aspects ensured that digital teaching could only develop step by step [16], [17]. The above-mentioned study results were only slowly being adopted in teaching practice. Many projects in digital teaching were carried out, but rarely evaluated.

Then came the COVID-19 pandemic and in a very short time teaching had to be converted almost completely to pure online teaching. In a one-off action, digital concepts were launched at universities and schools everywhere. Due to the short-term nature of the project, many different forms of digital teaching and examinations have been created. In contrast to earlier developments, this was less driven by institutional strategies and more by the de facto necessity and lack of alternatives. The actual design of teaching was strongly dependent on the existing resources, digital skills, time frame and the available technical infrastructure, which were heterogeneous at the various educational institutions.

The idea of making innovative projects from different locations visible was born at the beginning of the “Corona Semester”. It would have been a wasted potential not to make the digitisation push visible and to make the many different ideas visible at the educational institutions. However, here too, one challenge was how to make the individual projects evident as quickly as possible, independent of the very lagging review processes in the scientific world. We then decided to publish a focus issue with short contributions to project reports. 

What also caught us off guard was the very positive response of the special issue in the scientific community. A total of 128 contributions were submitted, which, together with the commitees “Digitisation – technology-supported learning and teaching” and “Interprofessional

training”, as well as the chief editors and Beate Hespelein, were very pleased with the results, but it also faced us with major challenges. We also had to break new ground in the review process and the selection of the corresponding contributions. In particular, we followed the idea that this issue should not be about completely finished research projects, but rather about the various activities and good practice concepts of the actors. Thanks to the joint efforts of all those involved, we have now succeeded in publishing the first of two parts of this special issue. The second part will be published on 28.01.2021.

In this first special issue, the many different articles will now be presented in the hope that the many projects and solutions will serve as inspiration for their own digital teaching. Digital teaching has found its way into all areas of training in medicine and the health care professions, which can be seen in the great variety of contributions. The areas of digital teaching, simulations and virtual patients existed before the COVID-19 pandemic, but now areas such as ethics, mentoring, communication have also been added and reveal new perspectives and challenges that are certainly interesting for all areas of higher education. Digital ideas are particularly in demand in skills training and practical examination formats. Special topics such as communication, examinations and ethics have posed particularly great challenges to teaching, as a large number of articles on these topics show. 

We would like to take this opportunity to thank all reviewers who have given us such active support at short notice. Without this unprecedented joint effort, the focus issues would not have been possible. This special issue is now the most voluminous edition in the history of JME.

We hope that this issue can give a lot of new impulses for the design of digital teaching and we also hope that the many different projects in digital teaching will also survive the COVID-19 pandemic. Last but not least, we also hope that digital education and training will enable tomorrow's health personnel to master crises such as the COVID-19 pandemic in the health system.

## Competing interests

The authors declare that they have no competing interests. 
